# Intrinsic Iron Release Is Associated with Lower Mortality in Patients with Stable Coronary Artery Disease—First Report on the Prospective Relevance of Intrinsic Iron Release

**DOI:** 10.3390/biom8030072

**Published:** 2018-08-09

**Authors:** Julia Ruhe, Christoph Waldeyer, Francisco Ojeda, Alev Altay, Renate B. Schnabel, Sarina Schäfer, Karl J Lackner, Stefan Blankenberg, Tanja Zeller, Mahir Karakas

**Affiliations:** 1 Department of General and Interventional Cardiology, University Heart Center Hamburg, 20246 Hamburg, Germany; juliapaolaruhe@gmail.com (J.R.); c.waldeyer@uke.de (C.W.); f.ojeda-echevarria@uke.de (F.O.); alevaltay@web.de (A.A.); r.schnabel@uke.de (R.B.S.); sar.schaefer@uke.de (S.S.); s.blankenberg@uke.de (S.B.); t.zeller@uke.de (T.Z.); 2 German Center for Cardiovascular Research (DZHK), Partner Site Hamburg, Lübeck, Kiel, 20246 Hamburg, Germany; 3 German Center for Cardiovascular Research (DZHK), Partner Site Rhein-Main, 55131 Mainz, Germany; Karl.Lackner@unimedizin-mainz.de; 4 Department of Laboratory Medicine, University Medical Center, Johannes Gutenberg University Mainz, 55131 Mainz, Germany

**Keywords:** iron release, coronary artery disease, biomarker, prognosis, hepcidin

## Abstract

Intrinsic iron release is discussed to have favorable effects in coronary artery disease (CAD). The aim of this study was to evaluate the prognostic relevance of intrinsic iron release in patients with CAD. Intrinsic iron release was based on a definition including hepcidin and soluble transferrin receptor (sTfR). In a cohort of 811 patients with angiographically documented CAD levels of hepcidin and sTfR were measured at baseline. Systemic body iron release was defined as low levels of hepcidin (<24 ng/mL) and high levels of sTfR (≥2 mg/L). A commercially available ELISA (DRG) was used for measurements of serum hepcidin. Serum sTfR was determined by using an automated immunoassay (). Cardiovascular mortality was the main outcome measure. The criteria of intrinsic iron release were fulfilled in 32.6% of all patients. Significantly lower cardiovascular mortality rates were observed in CAD patients with systemic iron release. After adjustment for body mass index, smoking status, hypertension, diabetes, dyslipidemia, sex, and age, the hazard ratio for future cardiovascular death was 0.41. After an additional adjustment for surrogates of the size of myocardial necrosis (troponin I), anemia (hemoglobin), and cardiac function and heart failure severity (N-terminal pro B-type natriuretic peptide), this association did not change (Hazard ratio 0.37 (95% confidence interval 0.14–0.99), *p* = 0.047). In conclusion, significantly lower cardiovascular mortality rates were observed in CAD patients with intrinsic iron release shown during follow-up.

## 1. Introduction

Iron is an essential component for the transport and utilization of oxygen and for appropriate mitochondrial function [1,2]. In ischemic as well as nonischemic systolic heart failure (HF), patients with preserved iron status have better prognosis than iron-deficient subjects, and iron supplementation has emerged as a guideline-endorsed therapy in deficient HF-patients [3]. 

Intrinsic systemic body iron release is defined as concomitance of low hepcidin and high soluble transferrin levels (sTfR) levels and mirrors a state of maximum bioavailability of iron in the circulation (low hepcidin), as well as increased utilization and high turnover of iron by demanding cells (high sTfR) [4]. 

Hepcidin is secreted by hepatocytes and orchestrates systemic iron metabolism [5]. Humans, as mammals in general, have no capacity of active iron excretion, therefore iron homeostasis is completely regulated through hepcidin [6]. The hepcidin production is feedback-regulated by iron levels: in the case of iron deficiency or increased iron needs, the suppression of hepcidin levels, i.e., low levels of hepcidin, causes an increase in dietary iron absorption in the duodenum, as well as iron release from macrophages [7]. After the cellular uptake of iron through the transferrin receptor on the cell surface, the plasma-bound transferrin receptor is released into serum by proteolytic cleavage [4]: accordingly, high circulating levels of sTfR reflect high turnover and increased utilization of circulating iron [8]. Importantly, sTfR levels are not affected by concomitant chronic disease and inflammation [9]. 

Here, we hypothesized that intrinsic iron release might be beneficial in coronary artery disease (CAD). We report (i) the prevalence of systemic iron release in patients with CAD, (ii) the rate of correlation of hepcidin and sTfR with various clinical and demographic factors, and (iii), its prognostic value in 811 patients with CAD beyond established and emerging cardiovascular risk factors.

## 2. Materials and Methods 

### 2.1. Study Population

Between June 1999 and March 2004, 3800 patients who underwent coronary angiography were recruited in the AtheroGene Study at the Department of Medicine II of the Johannes Gutenberg-University Mainz and the Bundeswehr-Zentralkrankenhaus Koblenz [10,11]. Surgery or trauma within the previous month, evidence of haemodynamically significant valvular heart disease, known cancer, known cardiomyopathy, febrile conditions, or use of oral anticoagulant therapy within the previous 4 weeks were exclusion criteria. 

Subjects with acute coronary syndrome (ACS), missing information on the cause of death, or missing information on the clinical presentation were additionally ruled out. This resulted in 2022 patients with stable CAD. Subsequently, subjects with missing samples or low sample volume, and missing laboratory measurements, were additionally excluded. This resulted in 811 subjects to be analysed in the present work. 

Informed consent was obtained by all patients. AtheroGene is in accordance with the Declaration of Helsinki and obtained approval by the Ethics Board of the Johannes Gutenberg-University Mainz and of the Physicians’ chamber of the State Rhineland-Palatinate (Germany) under the number 837.057.99.

### 2.2. Data Collection

Baseline information was obtained using standardized questionnaires and hospital charts. Regarding baseline medication, there was no difference between incident cases and noncases. Diagnosis of CAD was based on stenosis >30% in a major coronary artery revealed by cardiac catherization. Braunwald criteria were used in order to define unstable angina, while diagnosis of acute myocardial infarction (AMI) was based on cardiovascular guideline definitions [12]. All patients were followed-up until a mean of 4.0 years after discharge. For follow-up patients either presented at our clinic (87.2%) or were interviewed by telephone by trained medical staff. Information about the cause of death or clinical events was obtained from hospital or general practitioner charts using a mailed standardized questionnaire and adjudicated by two independent trained physicians. Deaths and respective causes (International Classification of Diseases, ICD-9 pos. 390-459, ICD-10 pos. I0-I99 and R57.0) were validated using death certificates. Cardiovascular death (death certificate) and/or nonfatal MI were used as endpoints.

### 2.3. Laboratory Methods

At baseline, blood was drawn before angiography in a fasting state under standardized conditions and stored at −80 °C until analysis. Levels of sTfR were measured using an automated immunoassay (Roche Cobas, Basel, Switzerland). The inter and intraassay coefficient of variation was 8.8% and 2.4%, respectively. Serum hepcidin was measured using a newly available immunoassay from DRG [13]. The inter and intraassay coefficient of variation was 9.7% and 8.7%, respectively. These measurements were performed from prior unthawed aliquots. The definition of systemic body iron release was based on hepcidin values <24 ng/mL and concomitant sTfR values ≥2 mg/L and derived from the overall cardiovascular disease cohort (*N* = 3274; respective median values). C-Reactive protein (CRP), N-terminal pro B-type natriuretic peptide (NT-proBNP,) troponin I, total cholesterol, high-density lipoprotein (HDL) cholesterol, and low-density lipoprotein (LDL) cholesterol measurements were done by routine methods in the participating hospitals [14–17]. All biomarkers were measured in a blinded fashion.

### 2.4. Statistical Methods

Patient population was described giving baseline characteristics, while the correlation of iron parameters with variables of interest is shown by calculating Spearman correlation coefficients. 

Hazard ratios for the outcome measure future cardiovascular mortality according to presence and absence of iron release were estimated by Cox regression models adjusted for potential confounders. Two adjusted models were constructed. Model 1 was adjusted for age (years), sex, hypertension, smoking status, diabetes, hyperlipidemia, body mass index (BMI), and model 2 additionally for hemoglobin, log(NT-proBNP), and log(troponin I). 

Statistics were calculated with R 3.2.3 [18]. All *p*-values below 0.05 were considered statistically significant.

## 3. Results

Table 1 shows the main sociodemographic and laboratory characteristics at baseline in 811 patients with CAD. Median age was 63.0 years, and participants were predominantly men (78.9%). While median sTfR was 2.2 mg/L, and median hepcidin was 23.2 ng/mL, prevalence of increased iron release at time of blood draw was 32.6%, as evidenced by concomitance of low hepcidin and high sTfR. 

In order to assess the correlation of sTfR and hepcidin with conventional and emerging cardiovascular risk factors, Spearman rank correlation coefficients (R) were calculated and yielded, if at all, moderate correlations (Table 2). Both iron parameters were significantly correlated with various risk factors, while most correlations were ≤0.10, and therefore negligible from a clinical point of view. A slightly stronger correlation was found between sTfR and NT-proBNP (R = 0.13; *p* < 0.001). Interestingly, both iron parameters showed no correlation with hemoglobin (sTfR: R = 0.02, *p* = 0.63; hepcidin: R = 0.01, *p* = 0.82).

During a median follow-up of 4.0 years, 27 patients (3.33%) experienced cardiovascular death. In order to assess whether systemic iron release is associated with lower rates of cardiovascular death during follow-up, Cox regression analyses were performed (Table 3). Hazard ratio (HR) regarding cardiovascular death was 0.41 (95% coefficient of variation (CI) (0.16–1.10)) in the basic model adjusted for sex, age, body mass index, smoking status, hypertension, diabetes, and dyslipidemia, which was formally nonsignificant (*p* = 0.076). Remarkably, this association did not change (HR 0.37 (95% CI 0.14–0.99), *p* = 0.047) after additional adjustment for common confounders of cardiovascular disease as of ((log) troponin), surrogate of systolic heart function ((log) NT-pro BNP), and anaemia ((log) hemoglobin). 

## 4. Discussion

In this study, we evaluated the prevalence of intrinsic iron release in patients with CAD and its prognostic relevance regarding future cardiovascular death in the midterm. To our best knowledge, this is the first study to evaluate the impact of intrinsic iron release in cardiovascular disease.

We report three major findings: first, almost one third of patients with stable CAD, 32.6%, showed increased intrinsic iron release at baseline. 

Secondly, correlation between both components of intrinsic iron release with conventional and emerging cardiovascular risk factors were negligible, if significant at all. Accordingly, both iron parameters showed no correlation with hemoglobin (sTfR: R = 0.02, *p* = 0.63; hepcidin: R = 0.01, *p* = 0.82). 

Thirdly, stable CAD patients with systemic iron release had significantly lower cardiovascular mortality rates, even after adjustment for surrogates of systolic heart function, size of myocardial necrosis, and anemia. Cardiovascular mortality rate over four years was 63% lower in those with intrinsic iron release (HR 0.37 (95% CI 0.14–0.99; *p* = 0.047)).

### 4.1. Pathophysiological Implications and Clinical Impact

The missing correlation between parameters of intrinsic iron release with hemoglobin indicates that anemia and iron homeostasis are distinct etiologies. This is supported by findings from recent randomized, placebo-controlled clinical trials: within the RED-HF trial, which mainly recruited HF-subjects with underlying CAD, treatment of anemia using darbepoetin alfa did not improve clinical outcomes during median follow-up of 28 months [19]. Moreover, within the FAIR-HF, in which two thirds of patients had CAD as underlying etiology of heart failure, the beneficial effects of intravenous iron supplementation were completely independent of hemoglobin levels as treatment with ferric carboxymaltose was beneficial to patients with and without anemia [20]. C-reactive protein, as surrogate marker for inflammation, showed the strongest correlation with both markers. This finding is in line with existing literature, showing that iron parameters mimic inflammatory status [21]. Nevertheless, the correlation is weaker than what was shown in previous reports for ferritin, iron, or transferrin saturation [22].

There are two main issues that need to be considered in relation to the CAD pathophysiology [23]: First, low hepcidin levels indicate absence of myocardial ischemia, since hepcidin seems to mirror subclinical and clinical myocardial ischemia and hypoxia [24]. Hepcidin is the key regulator of iron homeostasis. Low hepcidin levels guarantee high bioavailability of iron, while in contrast, elevated hepcidin levels inhibit the release of stored iron into the circulation [25]. In line, hemochromatosis type 2 gene mutations result in the complete absence of hepcidin and an early-onset form of iron-overload disease [26]. Moreover, the hepcidin-receptor ferroportin is highly expressed in duodenal enterocytes, as well as in macrophages from the reticuloendothelium, which in turn are involved in atherogenesis [27,28]. With respect to CAD, pathophysiological data premises that hepcidin might also mirror subclinical ischemia, so low levels of hepcidin might encode absence of myocardial ischemia [29]. In line with this finding, the working group of Kulaksiz proofed elevated levels of hepcidin within a rat model of acute hypoxia, and a rat model of AMI [30,31]. Isoda and coworkers confirmed these findings in a rat model of AMI and translated this to acute myocarditis patients [29]. Here, an abrupt increase of hepcidin up to 100 times was shown in human cardiomyocytes after myocardial infarction and acute myocarditis [29].

Secondly, the increased availability and turnover of iron, as mirrored by high sTfR levels, seems to be beneficial regarding the course of CAD. Iron is critically involved in the citric acid cycle [4]. It increases the expression of the enzyme aconitase by modulation of iron regulatory proteins. Experimental iron supplementation results in reduced glucose utilization and in increased mitochondrial adenosine triphosphate (ATP) formation. When bioavailability of iron is low, adverse glycolysis and lactate formation increases in order to compensate for decreased ATP production. This might enhance ischemic stress and apoptosis of cardiomyocytes [32]. Moreover, experimental data clearly show that during hypoxic conditions, mainly caused by underlying ischaemic disease, iron deficiency impairs the viability of cardiomyocytes [33]. In contrast, the availability of iron during hypoxic conditions is beneficial on genes associated with apoptotic activity in rat cardiomyocytes and also on skeletal myocytes. Iron seems to act as a protective agent, since levels of cellular atrophy decrease in hypoxic rat cardiomyocytes treated with iron-salt. More evidence comes from a recent study of cardiomyocyte-targeted deletion of iron-regulatory proteins (IRP) [34]. The activity of the mitochondrial iron-sulphur cluster-containing complex I was reduced in the left ventricles of IRP-mice compared with wild-type mice. In response to an acute dobutamine challenge, these mice were unable to increase left-ventricular systolic function and developed severe left-ventricular dysfunction and increased mortality after AMI. Interestingly, by intravenous iron injection mitochondrial respiratory capacity and inotropic reserve were restored, cardiac iron stores were replenished and adverse remodelling after AMI was attenuated in IRP-deleted mice. A recent clinical study points out that in subjects with CAD, high bioavailability of iron might result in improved remodelling [29]: by using serial cardiac MRI analyses, subjects with high (exogenously applied) iron bioavailability were shown to have a more pronounced decrease in infarct size (−10.3 ± 5.4% vs. −7.0 ± 8.4%, *p* = 0.050), a significant decrease in left-ventricular end systolic volume (71 ± 25 mL to 59 ± 25 mL, *p* = 0.002), and in addition a significant decrease in both, endocardial extent, and prevalence of transmural infarctions. These findings were irrespective of an iron deficiency (ID) status.

Our data show that intrinsic iron release is a wide-spread phenomenon in stable CAD, which is strongly associated with favourable outcome in the mid-term, independent of systolic heart function, size of myocardial necrosis, and anemia. The potential clinical significance of this project is substantial, regarding the fact that CAD remains a leading cause of morbidity and mortality worldwide. Approximately 450,000 people in the United States die from coronary disease per year, and the 2013 overall rate of death attributable to CVD was 222.9 per 100,000 Americans [35]. Based on our results and evidence from the literature, the favourable safety profile of intravenous iron supplementation, and the large evidence of anemia-independent efficacy of iron supplementation in systolic heart failure, we set up the multicentre, placebo-randomized, controlled CAYAN (comprehensive management of iron deficiency in myocardial infarction)-trial. The trial was registered and assigned the European Clinical Trials Database number 2015-005744-34.

Within this multicenter trial, patients with AMI and concomitant ID will be enrolled and randomly assigned to receive intravenous iron (ferric carboxymaltose) or saline (placebo). For the primary end point (change in left-ventricular ejection fraction from baseline to month 4), all patients will undergo cardiovascular magnetic resonance at baseline and at follow-up.

### 4.2. Strength and Study Limitations

The strengths of our study are the high standard of biobanking applied, and the fact that diagnosis of CAD was exclusively based on coronary angiography. Importantly, myocardial iron metabolism is a key component of the cardiac response to injury, especially in ischemic heart disease and involves ferroportin and l-type calcium channels. In this report, we assessed systemic iron body iron release which differs from myocardial iron metabolism. Furthermore, we used NT-proBNP as a surrogate for heart function, since routine echo was not applied within the protocol of this study. Moreover, like in most CAD populations, women are clearly underrepresented.

## 5. Conclusions

CAD patients with intrinsic body iron release showed significantly lower cardiovascular mortality rates during midterm follow-up. Meanwhile, this report on the prospective relevance of intrinsic iron release paved the way to the first controlled multicenter trial, randomizing CAD patients for intravenous iron supplementation versus placebo (European Clinical Trials Database number: 2015-005744-34). 

## Figures and Tables

**Table 1:** Baseline characteristics of the study patients.

*N*	811
Age (years) *	63.0 (55.0, 68.8)
Male sex (%)	78.9
BMI (kg/m²) *	27.2 (25.1, 30.1)
Smoker (%)	16.9
Diabetes (%)	22.1
Hypertension (%)	80.1
Hyperlipidemia (%)	79.9
History of MI (%)	45.3
Total Cholesterol (mg/dL) *	193 .0 (163.0, 222.0)
HDL-C (mg/dL) *	48.0 (41.0, 57.0)
LDL-C (mg/dL) *	120.0 (94.0, 147.0)
Troponin I (ng/mL) *	0.1 (0, 10)
NT-proBNP (pg/mL) *	148.4 (76.3, 365.4)
CRP (mg/dL) *^#^	2.3 (1.2, 4.7)
sTfR (mg/L) *	2.2 (1.7, 2.7)
Hepcidin (ng/mL) *	23.2 (15.4, 34.7)
Hemoglobin (g/dL) *	14.1 (13.1, 15.0)

BMI: body mass index, HDL-C: high-density lipoprotein-cholesterol, LDL-C: low-density lipoprotein-cholesterol, CRP: C-reactive protein, MI: myocardial infarction; NT-proBNP: N-terminal pro B-type natriuretic peptide; CRP: C-reactive protein; sTfR: soluble transferrin receptor. * Median 25th and 75th quartile cut-point. ^#^ Only available in a subset of patients.

**Table 2:** Spearman rank correlation coefficients.

	sTfR (*p*-Value)	Hepcidin (*p*-Value)
Male gender	−0.10 (0.005)	0.08 (0.025)
Age	0.05 (0.15)	−0.02 (0.62)
Smoking status	−0.03 (0.41)	−0.03 (0.41)
Diabetes	0.07 (0.038)	−0.02 (0.66)
Hypertension	0.01 (0.77)	0.05 (0.14)
History of MI	−0.04 (0.24)	−0.01 (0.75)
Hyperlipidemia	−0.04 (0.24)	−0.01 (0.75)
BMI	0.06 (0.073)	0.12 (<0.001)
Troponin I	0.10 (0.006)	0.05 (0.14)
NT-proBNP	0.13 (<0.001)	0.02 (0.66)
Hemoglobin	0.02 (0.63)	0.01 (0.82)
CRP	0.17 (<0.001)	0.24 (<0.001)
Creatinine	0.06 (0.074)	0.06 (0.08)
eGFR	−0.11 (0.002)	−0.02 (0.58)

**Table 3:** Association of systemic body iron release with cardiovascular (CV) death during follow-up (maximum 6.7 years).

	HR	95% CI	*p*-Value
Model 1	0.41	0.16–1.10	0.076
Model 2	0.37	0.14–0.99	0.047

HR: Hazard ratio, CI: confidence interval. Model 1: adjusted for age, sex, hypertension, smoking status, diabetes, hyperlipidemia, BMI. Model 2: Model 1 additionally adjusted for hemoglobin, log (NT-proBNP), log (troponin I).
